# *Bordetella pertussis* Infection Exacerbates Influenza Virus Infection through Pertussis Toxin-Mediated Suppression of Innate Immunity

**DOI:** 10.1371/journal.pone.0019016

**Published:** 2011-04-20

**Authors:** Victor I. Ayala, John R. Teijaro, Donna L. Farber, Susan G. Dorsey, Nicholas H. Carbonetti

**Affiliations:** 1 Department of Microbiology and Immunology, University of Maryland Medical School, Baltimore, Maryland, United States of America; 2 Department of Surgery, University of Maryland School of Medicine, Baltimore, Maryland, United States of America; 3 University of Maryland School of Nursing, Baltimore, Maryland, United States of America; Instituto Butantan, Brazil

## Abstract

Pertussis (whooping cough) is frequently complicated by concomitant infections with respiratory viruses. Here we report the effect of *Bordetella pertussis* infection on subsequent influenza virus (PR8) infection in mouse models and the role of pertussis toxin (PT) in this effect. BALB/c mice infected with a wild-type strain of *B. pertussis* (WT) and subsequently (up to 14 days later) infected with PR8 had significantly increased pulmonary viral titers, lung pathology and mortality compared to mice similarly infected with a PT-deficient mutant strain (ΔPT) and PR8. Substitution of WT infection by intranasal treatment with purified active PT was sufficient to replicate the exacerbating effects on PR8 infection in BALB/c and C57/BL6 mice, but the effects of PT were lost when toxin was administered 24 h after virus inoculation. PT had no effect on virus titers in primary cultures of murine tracheal epithelial cells (mTECs) in vitro, suggesting the toxin targets an early immune response to increase viral titers in the mouse model. However, type I interferon responses were not affected by PT. Whole genome microarray analysis of gene expression in lung tissue from PT-treated and control PR8-infected mice at 12 and 36 h post-virus inoculation revealed that PT treatment suppressed numerous genes associated with communication between innate and adaptive immune responses. In mice depleted of alveolar macrophages, increase of pulmonary viral titers by PT treatment was lost. PT also suppressed levels of IL-1β, IL-12, IFN-γ, IL-6, KC, MCP-1 and TNF-α in the airways after PR8 infection. Furthermore PT treatment inhibited early recruitment of neutrophils and NK cells to the airways. Together these findings demonstrate that infection with *B. pertussis* through PT activity predisposes the host to exacerbated influenza infection by countering protective innate immune responses that control virus titers.

## Introduction

In 2010 the California Department of Public Health declared a pertussis epidemic across California, the worst the state has seen in 63 years, with over 9400 cases and 10 infant deaths [1], [2]. The resurgence of pertussis or whooping cough in vaccinated populations poses a significant public health concern, especially for cases of mixed respiratory infections with viruses [3], [4]. Mixed respiratory infections can present with more severe disease, including acute bronchiolitis, viral pneumonia and infant respiratory distress syndrome (IRDS), resulting in loss of pulmonary function [5], [6], [7], [8]. Several pathogenic viruses, including adenovirus, rhinovirus and influenza virus, have been detected in the airways of patients with confirmed pertussis [9], [10], [11], [12], [13]. Infants under 4 months are at greatest risk for coinfection with respiratory syncytial virus (RSV), which can be fatal [6], [14], [15], [16], [17]. Analysis of sputum and nasal aspirates from acute and convalescent phase pertussis patients indicates that the rate of viral co-infection can be as much as 30% in adult populations and 16% in infants, and infection with more than one virus is common [1], [9], [12], [18]. However the actual rate of viral co-infection with *B. pertussis* is believed to be higher, but has been difficult to determine because these types of infections are frequently found by chance and often go undiagnosed or unreported [9], [19]. The high prevalence of viral infections and associated pathological conditions supports the theory that *B. pertussis* predisposes to such infections, possibly through the effects of its virulence factors [3], [5], [10].

Pertussis toxin (PT) is a multisubunit exotoxin produced exclusively by *B. pertussis* that ADP-ribosylates G proteins in mammalian cells to disrupt multiple G protein-coupled receptor signaling pathways [20], [21]. Recent studies on the role of PT during infection suggest that this toxin has long lasting effects on the immune system that could potentially benefit an ensuing viral pathogen [22], [23]. Using a mouse model of respiratory tract infection, we previously found that PT is required early during the bacterial infection [24]. Compared to a wild type infection, PT-deficient *B. pertussis* had reduced bacterial loads by 24 h post-inoculation. Administration of purified PT into the airways of mice prior to inoculation with the PT-deficient *B. pertussis* strain enhanced the bacterial infection, but not when administered 24 h post-inoculation. We also demonstrated that PT targets resident alveolar macrophages (AMs) to enhance the bacterial infection, since depletion of AMs allowed the PT-deficient *B. pertussis* strain to grow to wild type numbers [25]. A single dose of PT administered intranasally to mice modified the G proteins of AMs for up to 2 weeks, which was equivalent to the duration of the enhancing effect of PT treatment on the bacterial infection, demonstrating its long lived effect [25]. In addition, PT has been shown to inhibit early inflammatory responses in the respiratory tract, which reduces neutrophil recruitment in response to *B. pertussis* infection [26], [27], and PT stimulates inflammatory responses at the peak of infection by inducing Th1- and Th17-associated cytokines, including gamma interferon (IFN-γ) and IL-17 [28]. The toxin has also been shown to suppress levels of serum antibody to *B. pertussis* antigens after infection of mice [29], reduce expression of major histocompatibility complex class II molecules on the surface of human monocytes [30], and modulate expression of surface markers on dendritic cells [31]. We hypothesize that these effects, and those still unknown, allow PT to compromise host immune responses and may contribute to a reduced ability to combat a subsequent or concomitant influenza virus infection.

Influenza is a highly contagious respiratory infection that can cause significant morbidity and mortality [32], [33]. Many factors can affect the severity of influenza infection, including the virulence of the virus, immune status and age of the host, and whether a person smokes [34], [35]. Influenza virus can also synergize with other pathogens in the respiratory tract to induce more severe disease [36], [37], [38], [39]. During the 1918 influenza pandemic, around 40 million people died. Some deaths appeared to be due to viral pneumonia, however clinical and pathological evidence indicates that the vast majority of people succumbed to secondary bacterial pneumonia [40]. Animal studies have shown that influenza infection preceding a respiratory bacterial challenge can result in a life-threatening secondary pneumonia [36], [41], [42]. A number of mechanisms have been proposed to explain how influenza predisposes the host to superinfection with an unrelated or heterologous pathogen. Recently investigators have described how host responses associated with protection against influenza can sensitize the host to secondary bacterial infections by inhibiting antibacterial responses. IFN-γ induced in response to influenza inhibits AM function by reducing expression of the class A scavenger receptor MARCO, inhibiting bacterial clearance [43]. Type I IFNs induced by influenza can also negatively impact the host by impairing production of neutrophil chemoattractants KC and MIP-2 following secondary challenge with *Streptococcus pneumoniae*, resulting in inadequate neutrophil responses during the early phase of host defense against secondary bacterial infection [44]. Neutrophilic inflammation driven by the chemokine MIP-2 also appears to be a determinant for influenza superinfection with *Bordetella parapertussis* [42].

Influenza infection models in which the order of pathogen administration has been reversed, with the bacterial infection preceding influenza, have been less studied but indicate that the bacterial infection can have varied effects depending on the species. For example, proteases produced by *Staphylococcus aureus* and *Aerococcus viridans* enhance influenza replication and pathogenicity in mice by increasing cleavage of influenza virus HA, which is required for virus release [45], [46], and *Serratia marcescens* facilitates HA cleavage activation indirectly by generating plasmin from plasminogen [47]. In contrast, infection with *S. pneumoniae* prior to influenza has been shown to protect and improve survival against influenza challenge. Pretreatment of mice with a lysate of non-typeable *Haemophilus influenzae* (NTHi) that induces inflammation protected mice from a lethal infection with influenza A/Hong Kong/8/68 (H3N2) [48].

Treatment with whole cell *B. pertussis* vaccine (killed cells) renders mice resistant to mouse adenovirus and rabies virus, but is contraindicated for infections with influenza virus and RSV [49], [50], [51]. Studies examining mechanisms of interactions between live *B. pertussis* and other viral pathogens in the airways have been limited. Recently a group reported that infection with an attenuated strain of *B. pertussis*, expressing inactive PT, protects mice against highly pathogenic influenza A viruses by dampening the cytokine storm [52]. The present study establishes a model for investigating the interaction between *B. pertussis* and influenza virus and tests the hypothesis that PT activity sensitizes the host to exacerbated virus infection. We find that intranasal administration of *B. pertussis* or PT prior to influenza virus increases viral load at early and later stages of infection and also increases lung pathology and mortality associated with the viral infection, demonstrating that a respiratory bacterial infection can exacerbate a subsequent virus infection through the enzymatic activity of a virulence factor, PT, which targets host G protein-coupled signaling. We also find that PT suppresses early innate host responses necessary to control the viral infection. The exacerbation of respiratory viral infections by pertussis is a potentially important public health issue and underscores the need for pertussis vaccination for people of all ages.

## Results

### Effect of *B. pertussis* infection on subsequent influenza virus infection and the role of PT

**Figure 1:**
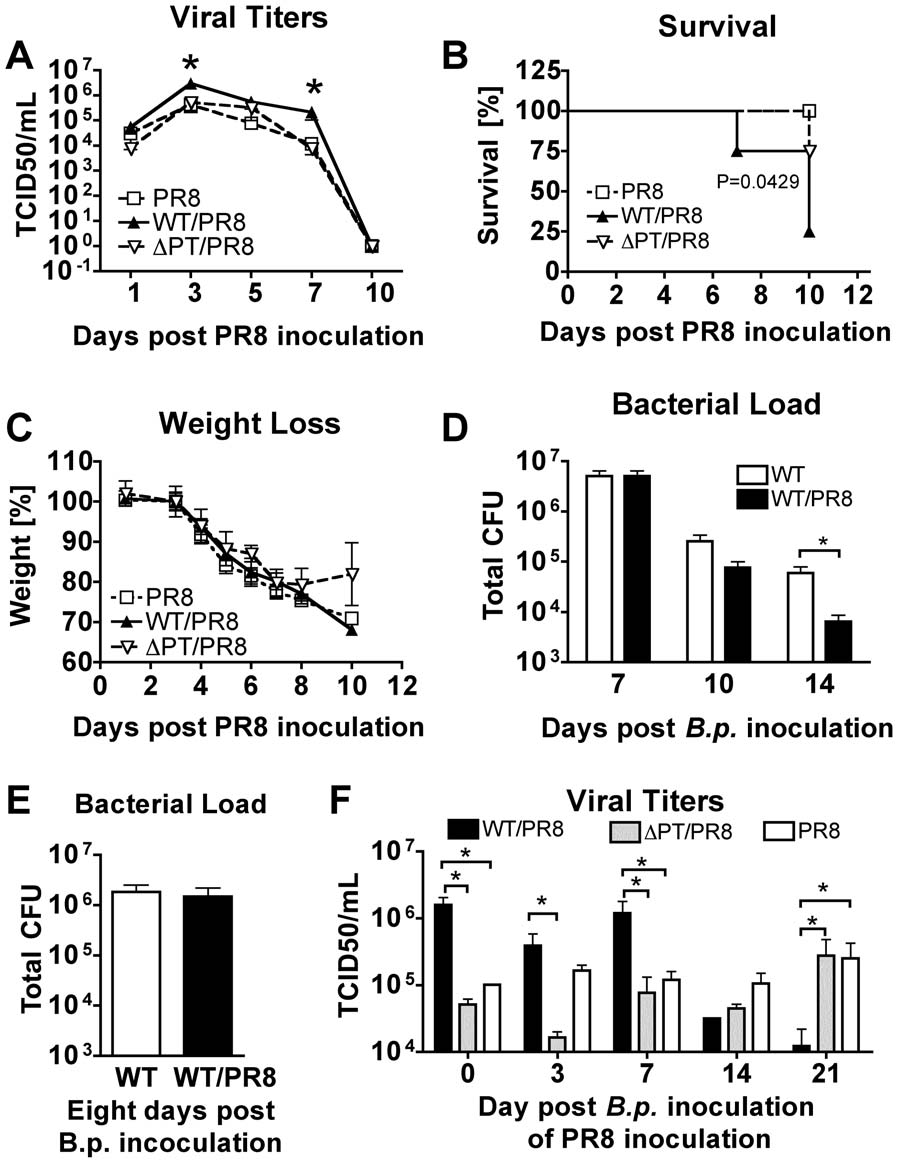
Influenza virus infection in *B. pertussis*-infected mice and effect of PT. BALB/c mice were inoculated with WT or ΔPT *B. pertussis* (5×10^5^ CFU) or PBS and seven days later infected with influenza virus PR8 (600 PFU). (A) Mean pulmonary viral titers, (B) survival rate, and (C) weight changes were assessed for 10 days post virus inoculation. (D, E) Bacterial loads in virus-infected and control mice at the indicated time points, when virus was inoculated (D) 7 days after bacterial inoculation, or (E) concomitantly with bacteria. (F) Mean pulmonary viral titers (day 6 post virus inoculation) in mice inoculated with WT or ΔPT *B. pertussis* and infected with influenza PR8 (600 PFU) at the indicated times post *B. pertussis* inoculation. n = 3–4 mice/treatment group/time point (4–5 mice for the last time point in A–C). *Significantly different from control (P<0.05).

To assess the effects of *B. pertussis* infection and the role of PT on secondary infection with influenza virus, BALB/c mice were inoculated intranasally with 5×10^5^ colony forming units (CFU) of *B. pertussis* (WT) or a mutant strain deficient in PT (ΔPT). Control mice were equivalently inoculated with PBS. Bacterial loads in the lungs assayed 4 days post-bacterial inoculation (n = 3) showed efficient infection (data not shown; WT = 4.4×10^6^ CFU, ΔPT = 5.6×10^5^ CFU). Seven days post-bacterial inoculation the mice were inoculated intranasally with 600 plaque-forming units (PFU) of a mouse-adapted influenza A/PR/8/34 (PR8) virus. Viral load in the lungs, weight loss (which is characteristic for influenza virus infection) and mortality were evaluated over the course of 10 days after virus inoculation (Fig. 1). Influenza-infected mice began losing weight by day 4 and showed signs of lethargy, wasting and ruffled fur. Weight loss was similar in all groups until day 8 (∼20%), but by day 10 the ΔPT-infected mice began to recover while the control group and the WT-infected mice continued to lose weight (Fig. 1C). On days 1, 3, 5, 7 and 10 post–viral inoculation, groups of mice (n = 3) were euthanized for determination of viral load from whole lung tissue. Viral load in the PBS control group peaked on day 3 post-inoculation at 3.9×10^5^ TCID50/ml and virus was cleared by day 10 (Fig. 1A). ΔPT-infected mice had a viral load profile similar to the PBS control group. However, viral load in WT *B. pertussis*-infected mice was significantly higher than that of the other groups on day 3 (3.3×10^6^ TCID50/ml, P = 0.0261) and on day 7 (2.4×10^5^, P = 0.047) post-inoculation (Fig. 1A). Furthermore, significantly greater mortality was observed in WT-infected mice (75%, P = 0.0429) than in PBS control mice (Fig. 1B). ΔPT infection enhanced mortality but to a lesser extent (25%, not significant vs. control mice). Infection with WT *B. pertussis*, but not with ΔPT, also increased overall lung pathology of the virus infection, as measured by total protein concentration in BAL fluid (data not shown). The *B. pertussis*-mediated increase in mortality associated with the virus infection was not due to the virus increasing bacterial loads, since there was no increase in WT *B. pertussis* numbers in virus-infected mice, either when virus was inoculated 7 days after the bacteria (Fig. 1D) or when virus was co-inoculated with the bacteria (Fig. 1E). *B. pertussis* ΔPT numbers were also unaffected by virus infection (data not shown). Collectively, these data demonstrate that prior infection with *B. pertussis* exacerbates influenza virus infection in a PT-dependent manner. The presence of PT in the WT *B. pertussis* infection has at least two effects on the influenza infection; (1) it increases virus titers in the airways; and (2) it enhances mortality.

### Longevity of *B. pertussis* exacerbation of influenza virus infection

To examine the longevity of *B. pertussis* exacerbation of influenza virus infection, mice were inoculated intranasally with WT or ΔPT *B. pertussis* (5×10^5^ CFU) or PBS as a control and groups of infected and control mice were inoculated with PR8 virus (600 PFU) at different times post-bacterial inoculation (day 0, 3, 7, 14 and 21). Mice were weighed daily to confirm and follow the viral infection (data not shown). Six days post-viral inoculation mice were euthanized for assessment of viral load in the lungs. As expected, levels of virus in PBS-treated control mice were comparable at all time points (∼10^5^ TCID50/ml) (Fig. 1F). Viral titers in ΔPT-infected mice were similar to those in PBS control mice except when virus was inoculated on day 3 post–bacterial inoculation, where the titers were lower (Fig. 1F). WT infection significantly enhanced virus growth (compared to ΔPT infection) when virus was inoculated on days 0, 3 and 7 post-bacterial inoculation and on days 0 and 7 compared to PBS control mice (Fig. 1F). The enhancing effect of WT infection was lost by day 14 post-bacterial inoculation and viral titers in WT-infected mice were significantly lower than in the other groups when virus was inoculated 21 days after bacterial inoculation (Fig. 1F), suggesting that the immune responses elicited by PT-producing *B. pertussis* during the later stages of the bacterial infection may be protective against virus infection. Together these data demonstrate that *B. pertussis,* through the action of PT, alters the lung environment in a manner that promotes virus growth early after bacterial infection and for a limited time (1–14 days).

### Effect of purified PT administration on influenza virus infection

**Figure 2:**
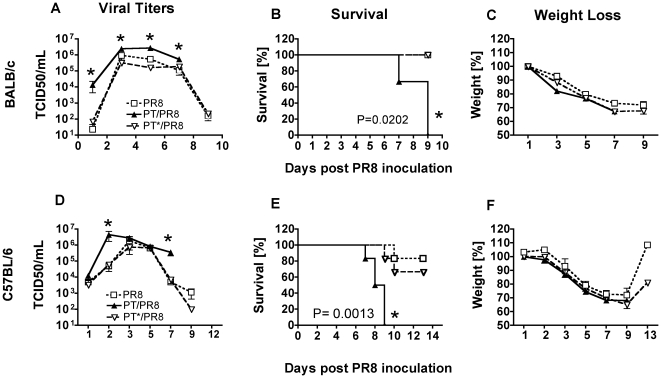
Effect of PT enzymatic activity on influenza infection in BALB/c and C57BL/6 mice. Mice were intranasally treated with 100 ng purified PT or PT-9K/129G (PT*) or PBS and infected with influenza virus PR8 (600 PFU) 24 h later. Mean pulmonary viral titers, survival rate and weight changes in BALB/c (A–C) and C57BL/6 (D–F) mice assessed for 9–13 days post virus inoculation. n = 3 mice/treatment group/time point (4–5 mice for the last time point for survival assessment). *Significantly different from control (P<0.05).

The results shown in Fig. 1 suggested that PT may be the factor responsible for *B. pertussis* exacerbation of influenza virus infection. Therefore, we tested whether purified PT, through its inhibitory activity on G protein signaling, could replicate the enhancing effect on virus titers in the airways independently of the bacteria. However, binding of the PT B oligomer (PTB) to cell surface molecules can elicit several intracellular signaling events independent of the enzymatic activity of the toxin A subunit [53]. Therefore, to determine if any observed differences in influenza virus titers caused by PT are due to its enzymatic activity or to the toxin's binding and signaling properties, mice were pretreated with active (PT) or inactive PT-9K/129G (PT*) toxin and infected with PR8 virus. PT* is structurally similar to active PT, but has 2 mutations in the A subunit that render it enzymatically inactive, which makes it an ideal control to distinguish between effects due to G protein ADP-ribosylation and other binding/signaling effects. Groups of BALB/c mice (n = 3) were inoculated intranasally with 100 ng PT or PT*, or an equal volume of PBS as a control, and 24 h later these mice were inoculated intranasally with 600 PFU of PR8. The mice were euthanized on days 1, 3, 5, 7 and 9 post-virus inoculation for assessment of virus infection and disease. As shown in Fig. 2A, control mice had a viral load that peaked on day 3 with a mean viral titer of 9.2×10^5^ TCID50/ml. Virus load decreased over the next few days and was cleared by day 9. PT*-treated mice had a viral load profile similar to the PBS-treated mice. In contrast to the controls, PT had an early enhancing effect on virus load. By day 1, PT treatment enhanced viral load by 140-fold over the control group (P = 0.018). The enhancing effect on virus titers persisted over the course of infection and the peak shifted from day 3 to day 5. PT treatment resulted in the death of all virus-infected mice by day 9, whereas none of the PT*-treated mice died by this time (Fig. 2B, P = 0.0202). However weight loss was not significantly different between groups (Fig. 2C). In a further investigation of the effect of PT on virus-associated mortality, groups of BALB/c mice (n = 8) were treated intranasally with 100 ng of PT or an equal volume of PBS as a control and 24 h later inoculated with a moderate dose (500 PFU) or a high dose (1500 PFU) of influenza virus PR8. No significant differences in weight loss were observed between groups (Fig. S1B and D). Mice pretreated with PBS and inoculated with 500 PFU of virus had a mortality rate of 50% by day 21 (Fig. S1A). In contrast, pretreatment with PT significantly reduced the survival rate, with no mice surviving past day 17 (P = 0.0421). At the higher dose of virus, none of the mice survived beyond day 10 (Fig. S1C); however the onset of death was 3 days earlier in the PT-treated group (P = 0.0286). Together these data demonstrate that, in addition to exacerbating other aspects of influenza infection and disease, PT significantly increases influenza mortality.

To determine whether the effect of PT in enhancing viral infection and disease could be reproduced in a different mouse background, we also examined PR8 infection in C57BL/6 mice treated with either PT or PT*. As shown in Fig. 2D, viral load peaked in the control and PT*-treated mice on day 3 post-inoculation and virus was cleared around day 9. PT treatment shifted the peak viral titer earlier to day 2 post-inoculation (4.6×10^6^ vs 6.1×10^4^ TCID50/ml, PT vs PBS), and delayed viral clearance on day 7. PT treatment significantly increased mortality of the virus infection over the other groups (P = 0.0013), and on day 9 all of the PT-treated mice were dead (Fig. 2E). In contrast, only one PT*-treated mouse died by day 9 (and 2 by day 13). Weight loss was not significantly different between groups over the first 9 days of virus infection (Fig. 2F). Together, these data demonstrate that only enzymatically active PT has an exacerbating effect on influenza disease. The effect was very similar in two different mouse strains, both of which experienced an early increase in viral titers and increased mortality as a result of PT treatment, ruling out the possibility that the effect was a peculiarity of a particular genetic background.

**Figure 3:**
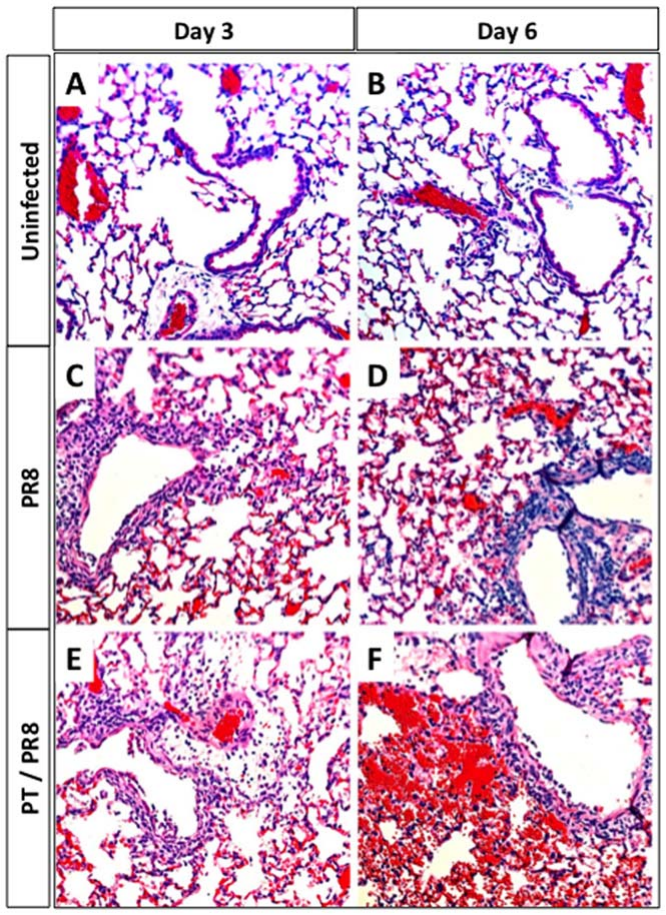
Histopathological examination of lungs from PT-treated and control mice infected with influenza virus. Lung sections of BALB/c mice inoculated with 100 ng of PT or with PBS and infected with influenza PR8 (600 PFU) 24 h later. Sections were prepared from (A–B) uninfected mice, or 3 and 6 days after virus inoculation from (C–D) PBS-treated or (E–F) PT-treated mice. Lungs were harvested and fixed immediately in 4% formalin. Sections were stained with hematoxylin-eosin and evaluated for density and location of cellular infiltrates. Sections include representative results from 3 mice/time point. Original magnification 10×. n = 3 mice/treatment group.

To assess changes caused by PT treatment on virus-induced lung pathology, total protein concentration in bronchoalveolar lavage (BAL) fluid of virus-infected, PT-treated, and control mice on days 2, 6 and 8 post-inoculation was measured by protein assay. The protein concentration was significantly higher in the BAL fluid of PT-treated virus-infected mice than in that of PBS-treated virus-infected mice or PT-treated uninfected mice on days 6 and 8 post-inoculation (Fig. S2). To further assess the impact of PT on lung pathology associated with influenza virus infection, lung sections from PR8-infected mice previously inoculated with PT or PBS were harvested on days 3 and 6 post-viral inoculation and analyzed by microscopy after staining with hematoxylin and eosin (Fig. 3). At day 3 post-inoculation, control virus-infected mice showed mild inflammation and sparse interstitial infiltrates (Fig. 3C) that increased on day 6 (Fig. 3D). PT-treated virus-infected mice were similar to PBS control mice on day 3 post-inoculation (Fig. 3C and 3E) but showed increased inflammatory cell infiltration, peribronchial cuffing and edema on day 6 (Fig. 3D and 3F). PT treatment alone without virus infection does not induce any significant lung pathology (our unpublished data). These data demonstrate that intranasal treatment with purified PT enhances the pathological manifestations of influenza, in addition to increasing the viral burden and mortality, replicating the effect of WT *B. pertussis* infection.

**Figure 4:**
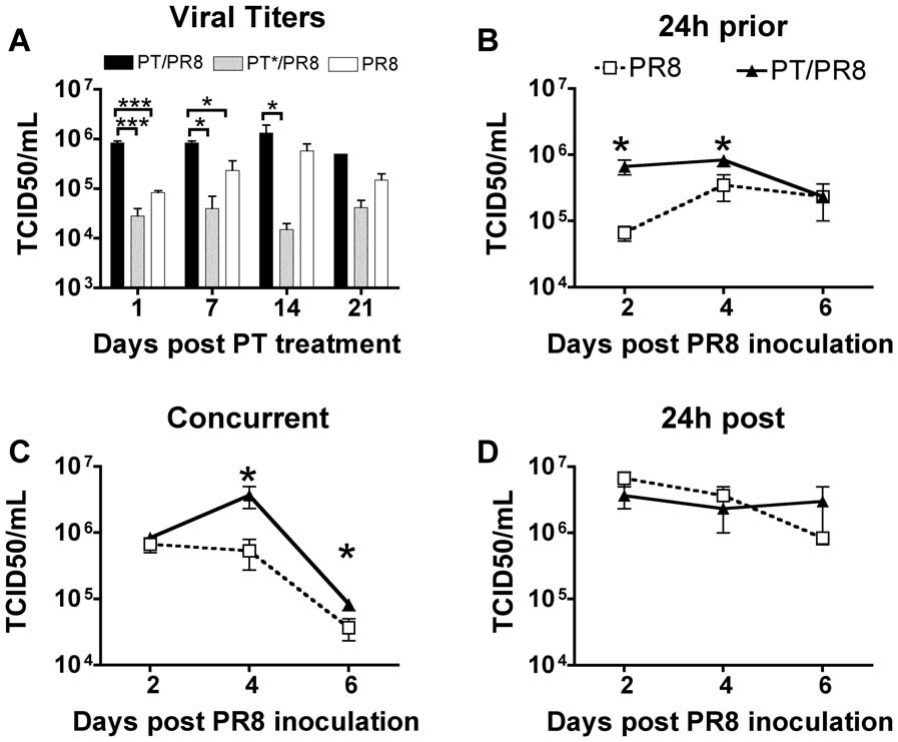
Effect of prior, concurrent and delayed PT treatment on influenza. (A) C57BL/6 mice were intranasally treated with 100 ng of PT, PT* or with PBS and infected with influenza PR8 (600 PFU) at the indicated times post PT treatment. Mean pulmonary viral titers were determined 2 days post-virus inoculation. (B–D) Mean pulmonary viral titers of mice treated with 100 ng of PT or PBS (control) 24 h prior (B), concurrently with (C), or 24 h post (D) infection with influenza PR8. Titers were determined 2, 4 and 6 days post virus inoculation. n = 3 mice/treatment group. *Significantly different from control (P<0.05).

We next assessed the longevity of the enhancing effect of PT, independent of the bacterial infection, in C57/BL6 mice. The results in Fig. 1F suggested that *B. pertussis,* through the activity of PT, provides influenza virus a window of opportunity that sensitizes the host for at least 7 days. Accordingly, we compared influenza virus infection in groups of C57BL/6 mice intranasally inoculated with 100 ng of PT or PT*, or with an equal volume of PBS as a control. At days 1, 7, 14 and 21 post-treatment, groups of mice (n = 3) were inoculated with 600 PFU of PR8. Lungs were harvested 2 days post-virus inoculation and viral titers were determined. This time point was chosen because it was the one at which PT showed the greatest effect in C57BL/6 mice (Fig. 2D). Fig. 4A shows that the mean virus titers in the lungs were significantly higher in PT-treated mice than in PT*-treated mice on days 1, 7 and 14 (and over the PBS control mice on days 1 and 7). There was still some enhancing effect of PT on day 21 post-treatment, though this was not quite significant (P = 0.087 vs. PT*-treated group). These results demonstrate that PT has a long-lasting enhancing effect on influenza virus titers that is dependent on its enzymatic activity but independent of the bacterial infection.

### Effect of prior or delayed administration of purified PT on influenza virus infection

Since the previous experiment established that administration of PT up to 7 days prior to influenza virus inoculation provided the virus a significant advantage over the PBS control (Fig. 4A), we tested whether PT treatment concomitant with or subsequent to virus inoculation also had an effect. Prior studies demonstrated the ability of PT to enhance *B. pertussis* respiratory tract infection when administered up to 14 days prior to bacterial inoculation, but not when administered 24 h after inoculation [24]. Therefore we tested whether the timing of PT inoculation has a similar effect on influenza viral infection by comparing the mean viral titers in the lungs of mice treated with 100 ng of PT intranasally 24 h before, concurrently with, or 24 h after virus inoculation. PT treatment was coordinated so that all mice were inoculated with influenza virus on the same day with the same dose (600 PFU). Lung tissue was harvested on days 2, 4, and 6 post-virus inoculation and mean pulmonary viral titers were determined. As seen in Fig. 4B, PT treatment prior to virus inoculation significantly enhanced viral load over the PBS control mice on day 2 (6.7×10^5^ TCID50/mL, P = 0.0231) and day 4 (8.3×10^4^ TCID50/mL, P = 0.047). However, the enhancing effect on viral load diminished by day 6. Co-inoculation of the toxin with the virus significantly increased viral titers on day 4 (3.7×10^7^ TCID50/mL, P = 0.0421) and day 6 post-infection (8.3×10^4^ TCID50/mL, P = 0.0470) (Fig. 4C). In contrast, no enhancing effect on viral titers was observed when PT was administered 24 h after virus inoculation (Fig. 4D). Together these data indicate that PT is capable of suppressing early events (0–24 h post-virus inoculation) that allows virus to replicate to higher levels in the mouse lungs.

### Effect of PT on influenza virus replication in vitro

**Figure 5:**
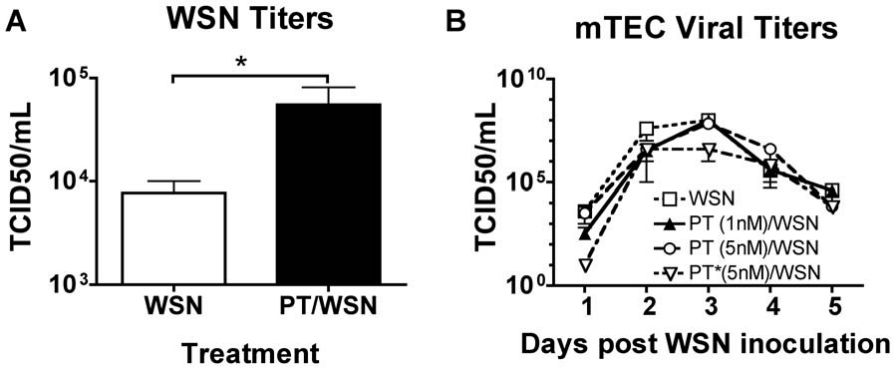
Effect of PT on influenza WSN virus infection in BALB/c mice and on mTECs. (A) Groups of BALB/c mice (n = 4) were pretreated with 100 ng of PT or an equal volume of PBS and 24 h later inoculated with 500 PFU of influenza WSN virus. Mean pulmonary viral titers were determined 3 days post virus inoculation. (B) mTECs (3 wells per group) were treated with 1 nM or 5 nM PT or 5 nM PT* for 24 h, or left untreated. The cells were then washed and infected with WSN virus at an MOI of approximately 0.001. After 1 h incubation, the inoculum was removed, and the cells were washed with medium and incubated at 37°C. At the indicated times, the infected-cell supernatant was sampled, and infectious virus titers were determined by TCID50.

Since our results thus far indicated that PT increases virus titers in vivo early after infection, we sought to address the question of whether PT directly enhances influenza virus replication in mouse airway cells. To this end we expanded our study to examine the replication of a recombinant H1N1 influenza virus strain A/WSN/33 (WSN) that has been shown to replicate efficiently in primary murine tracheal epithelial cell (mTEC) cultures over several days [54]. First we confirmed that PT still maintained an enhancing effect on this different influenza virus in mice. BALB/c mice (n = 3) were pretreated with 100 ng of PT or an equal volume of PBS and 24 h later inoculated with 500 PFU of WSN. Lungs were harvested 3 days post-inoculation and viral titers were determined. Treatment with PT significantly increased the pulmonary mean virus titer from 5.5×10^3^ to 7.7×10^5^ TCID50 (P = 0.0191) (Fig. 5A). Next mTEC cultures (n = 3) were treated with PT (1 nM or 5 nM) or PT* (5 nM) for 24 h, or left untreated. Cells were then washed with medium and infected with WSN at an MOI of approximately 0.001. After 1 h incubation, the inoculum was removed, and the cells were washed with medium and incubated at 37°C. MTECs were sampled on days 1, 2, 3, 4, and 5 post-infection and virus titers were determined. As seen in Fig. 5B, PT had little or no effect on viral titers up to and including day 5 post-infection at either concentration. This was similar for cells infected with PR8 (data not shown). Together these data indicate that PT does not enhance virus replication at the cellular level in vitro even though it can enhance viral titers in vivo, consistent with the idea that PT suppresses the immune response to allow the virus to replicate to higher levels.

### Effect of PT on the early type I interferon response to influenza

To better understand the potential influences PT has on the early phase of influenza virus infection, we analyzed the effect of PT treatment on the type I interferon (IFN) response to influenza virus infection. Type I IFNs IFN-α and IFN-β are cytokines with essential roles in innate viral immunity induced soon after influenza infection [55], [56]. Many viruses and some bacteria have acquired effective strategies to obstruct IFN activity [57], [58], [59], [60]. We hypothesized that PT could be working in a similar fashion to enhance influenza viral titers early after infection by preventing any of three events: IFN production, downstream signaling after IFN binding to its receptor, or expression of IFN-stimulated genes. To this end we assessed production of IFN-α in the airways of mice treated with PT and infected with influenza virus. BALB/c mice were treated intranasally with 100 ng of PT or given an equal volume of PBS as a control and inoculated with 600 PFU of PR8 24 h later. BAL supernatants were collected from infected animals at 1, 2, and 3 days post-virus inoculation and assayed by ELISA for the presence of IFN-α (Fig. S3A). Equivalent levels of IFN-α appeared in the BAL fluid of both groups of virus-infected mice at day 2 post-inoculation and declined for the control group at day 3. Interestingly, levels of IFN-α in PT-treated mice on day 3 were significantly higher than in control mice (322 vs. 122 pg/mL, P = 0.0212), suggesting that PT treatment sustained the expression of IFN-α. However, further examination of type I IFN expression in PT-treated and control virus-infected mice by a type I IFN bioactivity assay, which measures anti-viral activity of all type I IFNs produced, demonstrated that PT has no effect on the kinetics of type I IFN expression during 8 days post-virus inoculation (Fig. S3B). Therefore, PT does not appear to inhibit type I IFN production to increase early viral titers. In addition, we examined the level of tyrosine phosphorylation of STAT1 (signal transducer and activator of transcription) in A549 cells, widely used to study virus-IFN interactions [61], [62], to determine whether the observed effects of PT on virus titers might be due to altered type I IFN signaling. A549 cells were treated with PT (1 nM) for 24 h and stimulated with 1000 U of human IFN-α A/D. PT treatment resulted in modestly reduced tyrosine phosphorylation of STAT1 at 15 and 30 min post IFN-stimulation as judged by immunoblotting (Fig. S3C and D), however the phosphorylation level was equal during the peak of signaling at 45–60 min. These data indicate that inhibition of tyrosine phosphorylation of STAT1 by PT treatment is unlikely to account for the increased virus titers in mice. ISG15 is an ubiquitin-like protein rapidly induced in response to virus infection and IFN treatment [63]. Mice lacking ISG15 have increased susceptibility to influenza, herpes virus type 1, and Sindbis virus infection [64]. We therefore examined the expression pattern of soluble ISG15 and ISG15-protein conjugation in A549 cells with or without PT treatment (1 nM) and infected with influenza virus at an MOI = 1. Cell lysates were subjected to western blot analysis using anti-ISG15 antibody. Protein bands detected in control samples were similar to bands detected in PT-treated cells (Fig. S3E and F). Similar findings were made in cells treated with IFN (data not shown), together indicating that PT does not affect the expression of the IFN target protein ISG15 or its conjugation to other proteins. Together these data indicate that the type I IFN pathway does not appear to be a significant target for PT inhibition leading to increased viral titers.

### Microarray analysis of PT effects on influenza virus-induced pulmonary gene expression early after infection

Taking another approach to understand how PT treatment enhances viral titers during the early phase of influenza infection, we used gene expression analysis to provide a global view of the host response in lungs of infected mice. Whole genome expression microarray analysis was performed on total RNA isolated from lungs of mice treated intranasally with 100 ng of PT and infected with 600 PFU of influenza PR8 virus, compared to RNA from lungs of control mice treated with an equal volume of PBS and infected with an equal dose of PR8. Lungs from three mice for each treatment were harvested at 12 h and 36 h post virus inoculation (total n = 12). The fold change of virus-induced gene expression levels in PT-treated mice compared to control mice was averaged from the three mice per group, and averaged gene expression data was filtered using a cut-off value of 1.5-fold up- or down-regulation and a statistical significance of P<0.05. A total of 27 genes at 12 h and 51 genes at 36 h post-inoculation in PT-treated mice showed at least 1.5-fold difference in expression level compared to control infected mice (Table 1). The majority of PT-mediated changes in gene expression at 12 and 36 h post-infection were down-regulation, with 81% and 92% of the total, respectively (Table 1), indicating that PT has a significant suppressive effect on virus-induced gene expression.

**Table 1:** Numbers of genes differentially regulated in lungs of influenza virus-infected mice pretreated with PT versus control PBS-pretreated mice.

Time	Genes up^a^	Genes down^a^	Total
12 h	5	22	27
36 h	4	47	51

a Genes with a fold change greater than 1.5 (P<0.05) of PT-treated group over controls.

To characterize the functional consequences of gene expression changes associated with PT treatment and influenza infection, we performed pathway analysis of the gene expression data with Ingenuity Pathways Analysis. As shown in Table S1, this analysis revealed that PT treatment resulted in the most significant changes to antigen presentation, cell-to-cell signaling and interaction, gene expression, hematological system development and function, immune cell trafficking, inflammatory response and tissue morphology functions. These data show that PT alters the expression of a substantial number of genes that results in a decrease in pro-inflammatory and cell recruitment responses. Pathway analysis showed that, among the PT-affected genes, 5 pathways were significantly enriched at 36 h post virus inoculation, including several relating to some of the ascribed functions of AMs in response to influenza (Table 2). For example, the down-regulation effect of PT treatment is characterized by a suppression of genes related to communication between innate and adaptive immune cells, Fcγ receptor-mediated phagocytosis in macrophages and monocytes, antigen presentation and the role of hypercytokinemia/hyperchemokinemia in the pathogenesis of influenza pathways. Host genes with the highest fold changes between PT-treated and control virus-infected mice at 12 h (Table S2) and 36 h (Table S3) post-virus inoculation also support the hypothesis that innate immune responses and AMs are targets for PT suppression, and provide a basis for specific gene targets for future analysis. The lack of type I IFN-associated genes in this list confirmed our experimental observations from the previous section (Fig. S3). Collectively these data indicate that PT treatment prior to influenza infection suppresses a wide range of genes associated with numerous innate immune responses without specifically targeting one pathway over another. This subset of differentially regulated genes, however, relates closely to biological functions and cellular pathways of AMs, which play a critical role in regulating pulmonary immune responses to influenza viruses [65], [66], [67], [68], suggesting that PT reduces their capacity to respond to the virus and direct the ensuing innate and adaptive immune response.

**Table 2:** Pathways significantly affected by PT pretreatment in influenza virus-infected mice at 36 h post-inoculation.

Pathway Name^a^	p-value	Gene Count^b^
Communication between Innate and Adaptive Immune Cells	1.12E−05	5/83
Fcγ Receptor-mediated Phagocytosis in Macrophages and Monocytes	1.31E−03	3/91
Antigen Presentation Pathway	2.11E−03	2/29
Role of Hypercytokinemia/hyperchemokinemia in the Pathogenesis of Influenza	2.25E−04	3/31
Role of Pattern Recognition Receptors in Recognition of Bacteria and Viruses	3.35E−03	3/79

a Biological pathways analysis identified by Ingenuity Pathways Analysis library of canonical pathways that were most significant to the data set.

b Ratio of genes in a canonical pathway differentially regulated by PT treatment.

### Depletion of AMs eliminates the exacerbating effect of PT

**Figure 6:**
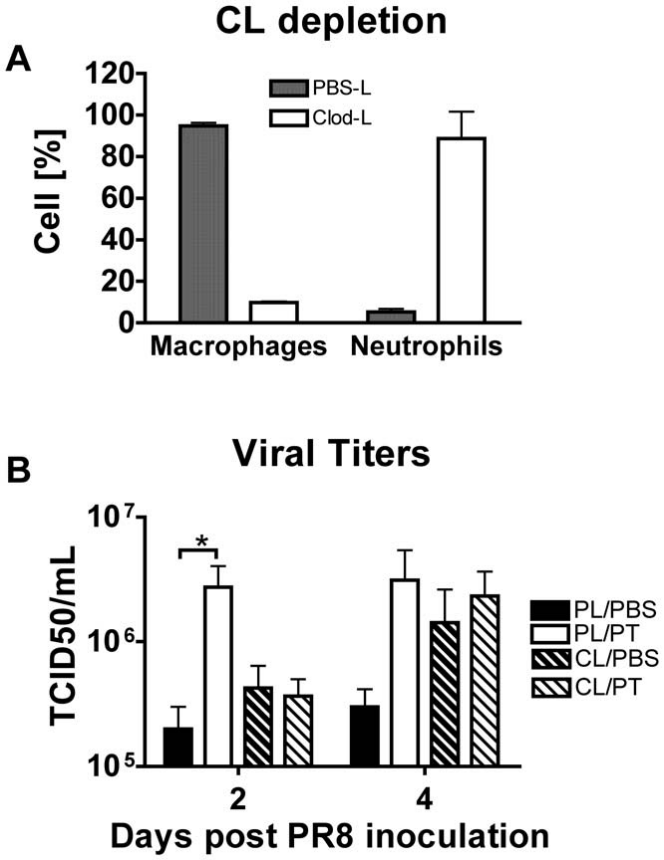
PT targets AM to enhance early viral titers and suppresses early pro-inflammatory airway responses. Groups of BALB/c mice were intranasally inoculated with 100 µL clodronate liposomes (CL) to deplete AM or with PBS liposomes (PL) as a control. (A) Percent of resident AM and neutrophils in the airways (BAL) two days after CL and PL treatment. (B) CL- and PL-treated mice were intranasally treated 2 days later with 100 ng of PT or an equal volume of PBS and infected with influenza PR8 (600 PFU) virus 24 h after PT/PBS treatment. Mean pulmonary viral titers were determined on days 2 and 4 post virus inoculation. n = 4 mice/treatment group. *Significantly different from control (P<0.05).

Since the microarray analysis implicated an association between PT treatment and AM dysfunction, we investigated the role of these cells by depleting them before PT treatment and infection with influenza virus. Depletion of AM was achieved by intranasal instillation of 100 µl of clodronate liposomes (CL) and control mice were administered an equal volume of PBS-liposomes (PL). CL administration resulted in an 86% reduction in AM numbers in BAL samples 2 days post-administration, compared to PL-treated mice (Fig. 6A). Groups of the CL- and PL-treated mice (n = 8) were then administered either 100 ng of PT or an equal volume of PBS and inoculated with the standard dose of influenza 24 h later. Lung tissues were collected for determination of viral titers at 2 and 4 days post virus inoculation (Fig. 6B). The mean pulmonary viral titer in PT-treated mice of the PL-group was 1.4 log higher on day 2 (P = 0.049) and 1 log higher on day 4 (P = 0.1324) compared to PBS-treated mice, typical of the exacerbating effect of PT on viral titers. Depletion of AMs abrogated the enhancement of viral titers by PT at both time points. We conclude that PT inactivation of the protective activity of AMs contributes to the increase in viral titers during the early phase of influenza infection.

**Figure 7:**
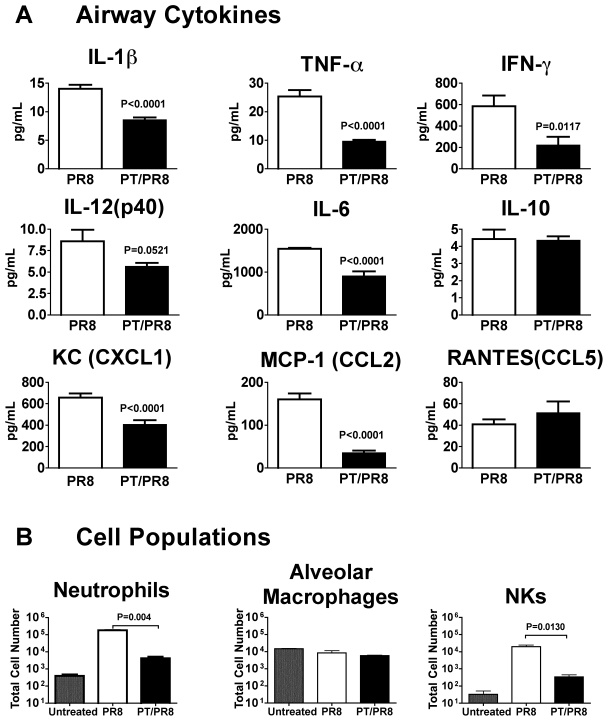
PT suppresses early pro-inflammatory responses in the airways. Groups of BALB/c mice were intranasally inoculated with 100 ng of PT or an equal volume of PBS as a control and infected with influenza PR8 (600 PFU) 24 h later. Cytokine concentrations and cellular recruitment was assessed two days after virus inoculation. (A) Lung (BAL) inflammatory cytokine levels assayed by multiplex bead array. (B) Numbers of lung immune cell populations in BAL fluid of PT-treated (PT/PR8) or PBS control-treated (PR8) mice infected with influenza virus (and in untreated mice). Neutrophils (CD11b^+^, Gr1^hi^), alveolar macrophages (CD11b^−^, CD11c^+^, F4/80^+^), and NK cells (NK1.1^+^). n = 4 mice/treatment group. Significant differences are indicated by P value.

AMs and lung epithelial cells coordinate the expression of many cytokines and chemokines to recruit and activate protective immune cells to the airways in response to influenza virus infection [66], [69]. Based on the pathway analysis findings from the microarray study, we hypothesized that PT would suppress recruitment of immune cells to the airways by reducing the expression of proinflammatory cytokines and chemokines. To test this we compared the cytokine/chemokine levels in mouse airways following PT or PBS treatment for 24 h and influenza virus infection. BAL samples were recovered 2 days post virus inoculation and assayed by multiplex array for levels of IFN-γ, IL-10, IL-12, IL-1β, IL-6, KC, MCP-1, RANTES and TNF-α (Fig. 7A). Levels of RANTES and IL-10 were similar for both virus-infected groups. However, the level of IFN-γ was 2.7-fold lower (P = 0.0117) in PT treated mice than in control mice and reduction in the levels of IL-1β, IL-6, KC, MCP-1 and TNF-αby PT treatment were highly significant (P<0.001). Next we examined the early cellular populations in the airways following PT or PBS treatment and influenza virus infection. BAL sample infiltrates were collected 2 days post virus inoculation and analyzed by flow cytometry (Fig. 7B). Consistent with the lower levels of cytokines, PT treatment resulted in greater than 1 log reduction in the number of infiltrating neutrophils (P<0.0001) and NK cells compared with that in PBS control mice (P<0.001). These data indicate that PT has an inhibitory effect on the early recruitment to the airways of cells that play a significant role in controlling virus replication and spread [70], [71], [72].

## Discussion

Individuals with a compromised immune system due to drug treatment, infection, old age and genetic disposition are at increased risk of infection with influenza. Early events during influenza infection dictate the outcome of the disease by containing and preventing excessive proliferation of virus in the respiratory tract, which is necessary for the adaptive immune response to mount an effective retaliation against the invading pathogen. *B. pertussis* is a bacterial airway pathogen that has specifically evolved a strategy to survive in the sterile environment of the human lungs by suppressing innate immune responses [23]. The virulence factor PT is very successful at suppressing innate immune responses, including cell recruitment, chemokine expression and antigen presentation, to promote bacterial survival in the respiratory tract. PT is unique from other bacterial toxins in that it effectively shuts down G protein-coupled receptor signaling, which is critical for important immune cellular function, and does this without killing the cells. Chemokines and cytokines that signal through G protein-coupled receptors are important for recruiting and activating effector cells, i.e neutrophils, monocytes, NKs, and T-cells, to the airways. Here we show that suppression of the innate immune response by PT is effective at sensitizing the host to a secondary respiratory pathogen. Infection with *B. pertussis* had a profound effect on an ensuing influenza virus infection. Mice inoculated with wild type *B. pertussis* (WT) and subsequently infected with influenza virus (PR8) had increased pulmonary viral titers at early, peak and clearance phases of infection, which was not observed in mice inoculated with an isogenic strain of *B. pertussis* deficient for PT (ΔPT). WT infection increased pulmonary viral titers by more than one log on days 3 and 7 post–virus inoculation and enhanced lung pathology associated with virus infection, as seen in the increase in total protein recovered from BAL samples and the destruction of lung architecture. We also observed that infection with WT bacteria increased influenza mortality 100% after 10 days of infection, which was not observed in ΔPT-infected mice, together suggesting a role for PT in disease exacerbation. Although we cannot formally rule out the possibility that mortality was due to influenza virus exacerbation of *B. pertussis* infection (rather than the other way around), this is very unlikely since *B. pertussis* infection in immunocompetent mice is rarely lethal, even at much higher bacterial loads than those seen in our experiments, and PT treatment was able to replicate the increased mortality effect.

Coadministration of *B. pertussis* and influenza virus increased viral titers, as did infections of *B. pertussis* administered 7 days prior to the virus. However, WT bacterial infection reduced influenza when virus was administered after 21 days, lowering the viral titers to almost undetectable levels and indicating that exacerbation of influenza infection by WT bacteria was time dependent. This was not seen in the ΔPT infection, indicating that PT may induce a switch in the immune response between 7 and 21 days post-bacterial inoculation that is beneficial for viral clearance. The host immune response to WT infection is significantly altered during this period. Following a respiratory challenge with *B. pertussis* the number of neutrophils, NK and NKT cells are elevated [73] as are the levels of IL-6, TNF-α, IFN-γ and IL-17 in the lungs. It is possible that one or a combination of these responses to the bacteria help clear the virus more efficiently. Some of these responses are indicative of Th1 immunity and are important for clearance of influenza. Together these results suggest that PT provides a window of opportunity that the virus can exploit for a period of time equivalent to its enhancing effects on the bacterial infection.

We also found that a single dose of purified PT administered intranasally was able to replicate the exacerbating effects of *B. pertussis* infection on influenza virus infection independent of the bacteria. Intranasal treatment with 100 ng PT followed by inoculation with PR8 7 days later significantly increased viral titers when compared to PBS control-treated mice, and also enhanced virus-associated lung pathology and increased mortality (PT treatment alone has no pathological effects on mouse lungs – our unpublished data). PT significantly increased the viral load over the course of 9 days of viral infection, especially early after inoculation and later after the peak, in both BALB/c and C57BL/6 mice. Since PT-treated mice had relatively high viral titers at the last time point sampled before death, it is not clear that these mice were able to clear the infection, which may have been a contributing factor to the enhanced mortality. Alternatively these mice could have died through the induction of a hyperimmune response (cytokine storm) which has been reported for more pathogenic viruses. The exacerbating effect of a single dose of PT on viral infection in C57BL/6 mice lasted at least 14 days, which is roughly equivalent to the duration of its enhancing effects for *B. pertussis* infection and the modifying effects observed on AM [25]. PT treatment alone does not induce inflammation or recruitment of monocytes or neutrophils to the airways (our unpublished data) and thus, without LPS stimulation from the bacteria, it is probable that the AMs do not turn over and remain modified in the airways and ineffective to respond to an invading pathogen. Since pretreatment and co-treatment of mice with PT (relative to virus inoculation) increased lung viral loads, but not PT treatment after virus inoculation, this indicated that PT is targeting a component of the early innate immune response with an important activity within the first 24 h of virus infection. PT also increased influenza viral titers of another influenza strain (WSN), demonstrating that this effect is not a peculiarity of PR8 infection. However, PT treatment of tracheal epithelial cell cultures did not significantly enhance virus replication in these cells, suggesting that PT does not directly increase cellular viral replication. Together these data led us to hypothesize that PT affects early immune responses, possibly anti-viral AM function, to enhance respiratory virus infection.

The early increase in viral titers was not due to PT inhibition of the type I IFN response. We had predicted that PT could increase viral titers in the airways by suppressing type I IFNs because AMs are the primary source of IFN-α in the airways in response to RNA viruses [74] and PT has been shown to inhibit expression of the cytokines TNF-α and IL-12 in monocyte-derived DC in a cAMP-dependent manner [75]. However we observed little effect on IFN expression in BAL samples of mice or cytokine signaling in vitro through the JAK/STAT signaling pathway. Nor did we observe a significant effect on the expression of the IFN-stimulated gene ISG15, indicating that PT does not inhibit activation of the type I IFN response nor does it interfere with the ability of ISGs to control virus replication. This implied to us that PT must be suppressing the innate immune response via another mechanism. To get at this question we used microarray technology to assess the effect of PT on immune responses to influenza in the whole lung, and pathway analysis revealed that PT inhibits communication between the adaptive and innate immune response, alters the expression of cytokines/chemokine in response to influenza and down regulates genes important for virus detection. Based on these results we hypothesized that PT was targeting AMs, as these pathways are associated with AM function. AMs are important for controlling virus replication and orchestrating the immune response, and they are an important source of chemokines and cytokines such as type I IFNs, IFN-γ, and TNF-α, which are required for recruitment and activation of effectors [76]. Additionally, AMs are responsible for phagocytosing infected epithelial cells [77] and antigen presentation to incoming CD8 cells, which ultimately clear the infection. Viral titer profiles and survival rates were similar in both BALB/c and C57BL/6 mice, which differ in the cytokine profile of their adaptive immune response, again suggesting that PT mediates its effects before the development of the adaptive response. We found that depletion of AMs resulted in abrogation of the PT enhancement of viral titers, supporting the hypothesis that PT targets AMs to increase virus titers. Depletion of these cells before a sublethal infection with 1918 HA/NA:Tx/91 virus was shown to result in increased virus growth, mortality and decreased expression of cytokines and chemokines in mice [67]. Therefore it is tempting to speculate that PT treatment is equivalent to depleting AMs prior to influenza infection. Indeed, at day 4 post-inoculation we found that PT treatment and AM depletion had equivalent enhancing effects on viral titers. However, lack of a significant increase in viral titers at day 2 by AM depletion in our experiments (Fig. 6B) would suggest that this interaction is complex. One possible explanation for the observed effect of PT (but not AM depletion) at day 2 is that PT targets other cells, possibly airway epithelia, in addition to AMs to increase viral titers.

We also found viral exacerbation to correlate with inhibition of neutrophil and NK cell recruitment early in the viral infection, as well as reduction in the levels of the chemokines KC (CXCL1) and MCP-1 (CCL2), which are chemoattractants for these cells. Levels of the cytokines TNF-α, IFN-γ, IL-6, IL-12 and IL-1β were also significantly reduced by PT treatment on day 2 post-viral inoculation. These data would suggest that PT inhibits cell recruitment and expression of Th1 cytokines early during influenza infection. TNF and IL-1 (which were both down–regulated by PT) produced by AM enhance chemokine-dependent neutrophil and T cell transmigration across influenza virus-infected epithelium [69], [78], [79]. Neutrophils play a critical role in controlling influenza disease as depletion of these cells has been shown to increase the mortality rate and pulmonary virus titers from the early to the late phase after infection [70]. By suppressing AM cytokine gene expression PT may be modulating the number and function of other effector immune cells required for viral clearance and controlling lung inflammation. However, in other studies we have found that PT treatment before virus inoculation increases the levels of several cytokines and chemokines later (day 4–8) in the virus infection, which may contribute to the increased lethality (Ayala and Carbonetti, unpublished data). Therefore PT may have a dual effect in exacerbating the virus infection – early inhibition of immune effector cell recruitment and later stimulation of damaging cytokine responses.

In conclusion, we have demonstrated that exacerbation of influenza occurs if the host is already infected with *B. pertussis.* While previous reports contraindicated pertussis vaccines for influenza, in this study we show for the first time that the effect of *B. pertussis* is attributable to the enzymatic activity of PT acting locally to suppress the innate immune response. If the mouse model is representative of human infections, the results of this study have broad implications, especially for communities where pertussis is on the rise. Undiagnosed pertussis infections could help spread an emerging virus or cause viral pneumonia to take hold in otherwise healthy individuals. Such a scenario may explain the observation of an out-of-season influenza outbreak in a nursing home where individuals had evidence of recent pertussis infection [10]. This would further emphasize the importance of pertussis vaccination for individuals at risk from influenza infections, including the elderly, for whom pertussis vaccines have only recently become available. Many immune processes are controlled or affected by G protein-coupled chemokine signaling, including pathogen detection, cellular activation, immune cell trafficking, and antigen presentation [80], [81], [82], [83], [84]. Whether the effects of PT are pathway-specific or the result of a more global inhibition has yet to be established. Future work will examine the effect of PT on virus-induced pathways, recruitment of effector cells and immune response gene expression. Importantly this paper demonstrates a mechanism by which *B. pertussis* can account for the high rate of coinfection with viruses. The emergence of viruses such as the 2009 pandemic influenza (pH1N1) virus in regions where *B. pertussis* is endemic underscores the need for more studies like this to examine the dynamics and mechanisms of interaction between *B. pertussis* and other respiratory pathogens so that better vaccination strategies or therapeutics can be developed to alleviate disease.

## Methods

### Bacterial Strains

The *B. pertussis* strains used for this study were streptomycin- and nalidixic acid-resistant derivatives of Tohama I and were produced as previously described [85]. The PT-deficient mutant strain (ΔPT) contains an in-frame deletion of PT genes and the wild-type (WT) strain is the parental strain that produces native PT [24]. The PT-9K/129G (PT*) strain of *B. pertussis* produces PT with 2 amino acid substitutions in the S1 subunit, rendering the toxin enzymatically inactive and thereby unable to ADP-ribosylate target G_i_ proteins [86]. *B. pertussis* strains were grown on Bordet-Gengou (BG) agar plates containing 10% defibrinated sheep blood and 400 µg/ml of streptomycin.

### Pertussis Toxin

PT and PT* were purified from *B. pertussis* culture supernatants. The proteins were purified over a fetuin affinity column as described by Kimura et al. [87]. The proteins were dialyzed against PBS to remove elution reagents, aliquoted and stored at −80^o^C. Protein concentrations were determined by BCA assay (Pierce) and confirmed by western blot. Activity of the toxins was determined by ADP-ribosylation assay as previously described [88].

### Viral Strains

The mouse-adapted influenza virus A/Puerto Rico/8/34 (H1N1)(PR8) was purchased from the American Type Culture Collection (ATCC; Manassas, VA) and grown in the allantoic fluid of 10-day old embryonated chicken eggs (Charles River Laboratories, Wilmington, MA) as previously described [89]. Recombinant influenza virus A/WSN/33 (H1N1) was provided by Andrew Pekosz (Johns Hopkins University). WSN was generated using a 12-plasmid rescue system in MDCK cells as described previously [90], [91].

### Mouse Infection

All mouse procedures were performed in accordance with the Public Health Service Policy on the Humane Care and Use of Laboratory Animals, and with protocol 0708002 approved by the University of Maryland, Baltimore Institutional Animal Care and Use Committee. Six-week-old female BALB/c or C57BL/6 mice (Charles River Laboratories) were used in our studies. Inocula of *B. pertussis* or PT were prepared in 50 µL PBS (pH 7.4) and mice were inoculated intranasally as previously described [28]. Control mice were treated with an equal volume of PBS. Following the bacterial infection or PT treatment, mice were challenged with PR8 or WSN in 50 µL PBS at specified times points. Weight of the mice was taken using a digital scale and overall appearance was recorded. Lung tissue was harvested for determining viral titers as previously described [92]. Briefly, mice were euthanized by carbon dioxide inhalation at specified times and the lungs were removed and homogenized in 2 ml of sterile PBS. The homogenate was clarified by centrifugation at 200×g for 5 min. The liquid phase was collected and stored at −70°C until assayed for viral titers. A minimum of 3 mice per group was used.

### Titration of virus by TCID50

Titers of viral stocks, lung homogenates and cellular supernatants were determined by the tissue culture infectious dose 50% assay (TCID50) as previously described [92], [93]. Briefly, half-log dilutions of samples in DMEM-5 containing penicillin and streptomycin were dispensed in a 96 well round bottom plate. Madin-Darby canine kidney (MDCK) cells were seeded on top. The following day media on the plates was changed for serum-free DMEM with 1% BSA and TPCK trypsin (Sigma-Aldrich) and incubated at 37°C for 4 days. Presence of virus was determined by performing hemagglutinin activity assays using chicken red blood cells (CRBCs). A suspension of 0.5% of CRBC was added to all the wells and the agglutination pattern was recorded after 1 h incubation at 4°C. The TCID50 was reported as the reciprocal of the dilution in which 50% of the infected wells were positive for virus calculated by the Reed-Muench method.

### Protein Concentration of BAL Samples

Mice were euthanized by carbon dioxide inhalation and dissection was performed to expose the trachea and lungs. A 20-gauge blunt-ended needle was inserted into a small incision towards the top of the trachea and tied in place with surgical thread. BAL was performed by flushing the lungs two times with 0.7 ml of sterile PBS. Total protein concentration recovered in the BAL fluid was measured by BCA assay (Pierce).

### Lung Histology

For histopathological analyses of lungs, mice were euthanized by carbon dioxide inhalation, trachea were exposed, and lungs were inflated and fixed with 4%-buffered formalin. The lungs were embedded in paraffin wax, sectioned and stained with H&E by the Pathology Core Facility (University of Maryland at Baltimore), and analyzed by light microscopy for density, composition, and location of inflammatory infiltrates from 3 mice per time point per group.

### mTEC Infection

mTEC cultures were prepared and kindly provided by Andrew Pekosz (Johns Hopkins University). Cells were maintained as previously described [94]. Cells in the apical chamber were treated with PT or PT* in DMEM containing 0.5% BSA and penicillin-streptomycin for 24 h. Apical cells were then infected with 3,600 PFU of virus diluted in warm DMEM with 0.5% BSA containing penicillin-streptomycin at a multiplicity of infection (MOI) of approximately 0.01. The cells were incubated with virus at 37°C for 1 h, the inoculum was removed, and cells were washed three times with DMEM containing penicillin-streptomycin. After washing, DMEM containing penicillin-streptomycin was placed in the apical compartment. Apical supernatants were collected at the indicated times postinfection and stored at 70°C.

### IFN bioassay

BAL samples were acidified to a pH 2 and incubated at 4°C overnight to inactivate any input virus as well as acid-labile IFNs and other cytokines. Samples were then neutralized to pH 7 with NaOH. Serial dilutions of each sample were added to mouse fibroblast cells (L929) in 96-well plates and incubated for 24 h. Encephalomyocarditis virus (EMCV) was then added at an MOI of 5, and the cultures were incubated for 48 h before scoring the plates for cytopathic effect (CPE). CPE was visualized by light microscopy and BAL-treated cells were scored as the dilutions of sample giving 50% protection from virus as compared to a standard curve of IFNβ-treated cells.

### Immunoblot analysis

A549 cell lysates were prepared by direct lysis in 1x Laemmli buffer. Proteins were separated by SDS-polyacrylamide gel electrophoresis (12% gel) and transferred onto nitrocellulose membranes (Bio-Rad). The blots were probed with: monoclonal mouse antibody specific for Phospho-Stat1(Tyr701) (Cell Signaling), rabbit anti-ISG15 antibody and monoclonal mouse antibody against β-actin (BD). Horseradish peroxidase-labeled secondary antibodies were used to detect primary antibodies and a chemoluminescence detection system (Amersham ECL Plus) was employed to develop the membrane. Signal quantification was measured from exposed film utilizing GelEval software to measure band intensity.

### RNA preparation

Total RNA was extracted from whole lung tissue using the phenol-chloroform method. Samples were homogenized in 1 ml of RNA Stat-60 (Tel-Test, Inc.). Subsequently, 200 µl of chloroform was added to each preparation, and the sample was centrifuged at 13,000 g for 15 min at 4°C. The aqueous phase was transferred to a 1.5-ml tube containing 500 µl of isopropanol, and the samples were stored at −20°C overnight. The samples were centrifuged at 13,000 g for 15 min at 4°C, and the supernatants were removed from the RNA pellets. The RNA pellets were washed twice with 80% ethanol and centrifuged, and ethanol was removed from the pellets. The samples were dried with a DNA Speedvac (Savant) at a low temperature for 15 min until the pellets became transparent. Each pellet was resuspended in 50 µl of nuclease-free H_2_O and placed in a 65°C water bath for 30 min. RNA samples were further cleaned up using the Qiagen RNeasy Mini Kit according to manufacturers protocol.

### Microarray Expression Profiling

Mouse gene expression was examined with the GeneChip Mouse Gene 1.0 ST Array (Affymetrix). RNA quality control, sample labeling, GeneChip hybridization and data acquisition were performed at the Biopolymer/Genomics Core Facility at the University of Maryland School of Medicine. Quantity and purity of the RNA samples was determined by examining the 260/280 ratio (Nanodrop, Thermo Scientific, Worcester, MA) and via analysis on the Experion RNA StdSens Analysis kit (Bio-Rad, Hercules, CA). One hundred nanograms of total RNA per sample was reverse transcribed to cDNA, converted and amplified to antisense cRNA and labeled with biotin in an in vitro transcription reaction according to the manufacturer's protocol (Affymetrix, Santa Clara, CA). Targets were hybridized to Affymetrix Mouse Gene 1.0 ST Array, which contains approximately 27 probes spread across the length of 28,853 genes. All arrays were scanned at the same time on the Affymetrix Gene-Chip Scanner 3000G and the resulting .cel files were used in the analysis.

The input files were normalized with full quantile normalization [95]. For each input array, for each probe expression value, the array ith percentile probe value was replaced with the average of all array ith percentile points. Next, the 906,151 probes were transformed into analysis values. Probes with a GC count less than 6 and greater than 17 were excluded from the analysis. The intensity values for probes included in the analysis were transformed by taking the Natural Logarithm of 0 plus the probe score. Probes were stratified by CG content and defined in the MouseGene10ST_antigenomic.bgp file. Each probe score was corrected for background by subtracting the median expression score of background probes with similar GC content. Expression scores for each probe-set were defined as the median of the probe expression scores. Differential gene expression was determined using analysis of variance (XRay v3.98; Biotique Systems, Reno, NV). Because of the large number of statistical tests performed, a False Discovery Rate (FDR) correction was performed to correct p-values. We defined a differentially expressed gene as one with a 1.5 fold change from control and an FDR-corrected p-value of less than 0.05. Data were analyzed through the use of Ingenuity Pathways Analysis (Ingenuity® Systems, www.ingenuity.com). The Functional Analysis identified the biological functions and/or diseases that were most significant to the data set. Molecules from the dataset that met the 1.5 fold change cutoff and P = 0.05 and were associated with biological functions and/or diseases in Ingenuity's Knowledge Base were considered for the analysis. Right-tailed Fisher's exact test was used to calculate a p-value determining the probability that each biological function and/or disease assigned to that data set is due to chance alone.

Canonical pathways analysis identified the pathways from the Ingenuity Pathways Analysis library of canonical pathways that were most significant to the data set. Molecules from the data set that met the 1.5 fold change cutoff and P = 0.05 and were associated with a canonical pathway in Ingenuity's Knowledge Base were considered for the analysis. The significance of the association between the data set and the canonical pathway was measured in 2 ways: 1) a ratio of the number of molecules from the data set that map to the pathway divided by the total number of molecules that map to the canonical pathway is displayed; 2) Fisher's exact test was used to calculate a p-value determining the probability that the association between the genes in the dataset and the canonical pathway is explained by chance alone.

### Cytokine measurements

Mice were euthanized and BAL was performed as described above. Resident cells were spun out by centrifugation 3 times at 13,000 g for 1 min. The supernatants were stored at −80°C, and multiplex cytokine array (Luminex 100 System) was performed for IL-1β, IL-2, IL-6, KC, IL-10, IL-12 (p40), IFN-γ, TNF-α, MCP-1, and RANTES at the UMB Cytokine Core Laboratory.

### Flow cytometric analysis

BAL samples were harvested as described above and centrifuged at 13,000×g for 1 min to recover airways cells. Cells were washed with staining buffer (1% FBS in PBS) and immunostained with fluorescently labeled antibodies (eBioscience) for CD11b (Clone M1/70), CD11c (Clone N418), Gr-1 (Clone RB6-8C5), and NK (Clone 14B11), for 30 min at 4°C and then washed twice in staining buffer. Cell samples were then fixed with 4% paraformaldehyde for 15 min at room temperature. Events were collected on a LSRII flow cytometer (Becton Dickinson) driven by FACSDiva software (Becton Dickinson), and analyzed using FlowJo software (Tree Star). Data were collected for 10,000 viable cells selected by forward and side scatter. Cellular profiles were characterized accordingly: AM (CD11c^hi^, CD11b^−^, F4/80^+^), Neutrophils (GR1^+^, CD11b^+^, CD11c^−^), NK (NK^+^).

### Statistical Analysis

All statistical analysis was performed using GraphPad Prism version 4 for Macintosh (GraphPad Software, San Diego, California, USA). Data were expressed as the mean ± s.d. Statistical significance of differences between experimental groups was determined using the student's t-test to compare two normally-distributed samples or ANOVA to compare multiple samples. Kaplan-Meier log-rank test was used for survival analyses. P values >0.05 were considered not to be significant. All data shown are representative of at least two independent experiments,

## Supporting Information

Figure S1 **Effect of PT treatment on survival rates of mice infected with influenza.** Groups of BALB/c mice were intranasally treated with 100 ng of PT and 24 h later inoculated with influenza PR8. (A) Survival of mice inoculated with a moderate (500 PFU) or (C) large (1500 PFU) dose of influenza virus. (B, D) Weight loss of mice in panel A and C respectively. n = 8 mice/treatment group *Significantly different from control (P<0.05).
(TIF)

Figure S2 **Effect of PT treatment on BAL protein concentration in influenza virus-infected mice.** Groups of BALB/c mice were intranasally treated with 100 ng of PT (control mice were treated with PBS) and 24 h later inoculated with influenza PR8 (600 PFU). BAL fluid was recovered from these mice on days 2, 6 and 8 post-virus inoculation and total protein concentration was determined. n = 4 mice/treatment group *Significantly different from control PR8-infected mice (P<0.05); **P<0.01.
(TIF)

Figure S3 **PT does not suppress type I IFN responses.** (A,B) BALB/c mice (n = 3) were treated with 100 ng of PT or an equal volume of PBS and infected with influenza PR8 (600 PFU) 24 h later. BAL fluid was recovered and assayed by (A) ELISA for IFN-α or (B) type I IFN bioassay at the indicated times post virus inoculation. *Significantly different from control (P<0.05). (C) Immunoblot analysis of lysates from A549 cells pretreated with or without 1 nM PT for 24 h and stimulated with 1000 U human IFN A/D and probed with anti-phospho-STAT1 (Tyr701) antibody. (D) Normalized pY-STAT1 band intensities. (E) Immunoblot analysis of lysates from A549 cells treated with or without 1 nM PT for 24 h and infected with influenza PR8 (MOI = 1) and probed with anti-ISG15 antibody. (F) Normalized ISG15 band intensities. Immunoblots are representative of two experiments. *Significantly different from control (P<0.05).
(TIF)

Table S1 Biological functions significantly affected by PT pretreatment in influenza virus-infected mice.
(PDF)

Table S2 Genes differentially-regulated by PT pretreatment in influenza virus-infected mice at 12 h post-inoculation.
(PDF)

Table S3 Genes differentially-regulated by PT pretreatment in influenza virus-infected mice at 36 h post-inoculation.
(PDF)
