# Supplementary material for: Bordetella pertussis Infection Exacerbates Influenza Virus Infection through Pertussis Toxin-Mediated Suppression of Innate Immunity
Source: PLoS One. 2011 Apr 20;6(4):e19016. doi: 10.1371/journal.pone.0019016 (PMC3080395; doi:10.1371/journal.pone.0019016)
Supplement: Table S1 — Biological functions significantly affected by PT pretreatment in influenza virus-infected mice. (PDF) [file pone.0019016.s004.pdf]

Table S1. Biological functions significantly affected by PT pretreatment in influenza virus-infected mice, ranked by gene count.

12 h			36 h		
**Biological Function[a]**	**Gene Count[b]**	**p-value**	**Biological Function[a]**	**Gene Count[b]**	**p-value**
Hematological System Development and Function	16	2.90E-05 - 4.73E-02	Hematological System Development and Function	24	8.43E-11 - 3.79E-02
Inflammatory Response	12	6.36E-04 - 4.78E-02	Inflammatory Response	23	8.60E-12 - 3.79E-02
Cell-To-Cell Signaling and Interaction	12	2.81E-05 - 4.73E-02	Cell-To-Cell Signaling and Interaction	21	6.65E-09 - 3.42E-02
Cellular Development	11	2.90E-05 - 3.72E-02	Inflammatory Disease	19	9.70E-13 - 2.98E-02
Cell Death	11	3.08E-05 - 4.36E-02	Cellular Movement	19	1.36E-12 - 3.42E-02
Cellular Growth and Proliferation	10	2.86E-05 - 4.57E-02	Immune Cell Trafficking	19	8.43E-11 - 3.42E-02
Tissue Morphology	10	1.51E-04 - 4.78E-02	Tissue Development	18	1.30E-07 - 3.79E-02
Immunological Disease	9	1.49E-06 - 4.99E-02	Hematopoiesis	17	7.00E-08 - 3.42E-02
Gene Expression	8	3.45E-07 - 3.90E-02	Immunological Disease Connective	16	2.37E-11 - 3.79E-02
Hematopoiesis	8	1.71E-04 - 4.57E-02	Organismal Survival	16	6.16E-06 - 4.56E-03
Inflammatory Disease	7	4.13E-04 - 4.36E-02	Cellular Function and Maintenance	14	1.12E-06 - 3.42E-02
Skeletal and Muscular System Development and Function	7	1.51E-04 - 4.36E-02	Antigen Presentation	13	2.38E-05 - 3.06E-02

[a] Functional analysis of changes in statistically significant gene expression was done using Ingenuity Systems Pathway Analysis (IPA) software.

[b] Genes with a fold change greater than 1.5 or more (P<0.05) of experimental group over controls.
