# Supplementary material for: Bordetella pertussis Infection Exacerbates Influenza Virus Infection through Pertussis Toxin-Mediated Suppression of Innate Immunity
Source: PLoS One. 2011 Apr 20;6(4):e19016. doi: 10.1371/journal.pone.0019016 (PMC3080395; doi:10.1371/journal.pone.0019016)
Supplement: Table S2 — Genes differentially-regulated by PT pretreatment in influenza virus-infected mice at 12 h post-inoculation. (PDF) [file pone.0019016.s005.pdf]

Table S2. Genes differentially-regulated by PT pretreatment in influenza virus-infected mice at 12 h post-inoculation.

Symbol	Gene Annotation [a]	Fold Change	P-value	Accession [b]
PER2	period homolog 2 (Drosophila)	1.91	1.24E-05	NM_011066
NME2	non-metastatic cells 2, protein (NM23B) expressed in	1.71	1.29E-06	NM_001077529
PER1	period homolog 1 (Drosophila)	1.71	4.09E-03	NM_011065
ADAMTS9	ADAM metallopeptidase with thrombospondin type 1 motif, 9	1.61	4.89E-03	NM_175314
ALAS2	aminolevulinate, delta-, synthase 2	1.61	8.74E-05	NM_009653
SLFN12	schlafen family member 12	1.61	2.87E-04	AF099974
TEF	thyrotrophic embryonic factor	1.51	4.50E-02	NM_017376
TNNC1	troponin C type 1 (slow)	1.51	1.52E-02	NM_009393
AHR	aryl hydrocarbon receptor	-1.51	4.20E-09	NM_013464
CCRL2	chemokine (C-C motif) receptor-like 2	-1.51	4.62E-04	NM_017466
HCLS1	hematopoietic cell-specific Lyn substrate 1	-1.51	3.62E-02	NM_008225
PLEK	pleckstrin	-1.51	1.09E-02	NM_019549
PTPN6	protein tyrosine phosphatase, non-receptor type 6	-1.51	8.52E-07	NM_013545
SOX18	SRY (sex determining region Y)-box 18	-1.51	6.02E-03	NM_009236
SPP1	secreted phosphoprotein 1	-1.51	5.49E-03	NM_009263
VIPR2	vasoactive intestinal peptide receptor 2	-1.51	1.08E-04	NM_009511
ABCG1	ATP-binding cassette, sub-family G (WHITE), member 1	-1.61	2.49E-05	NM_009593
GJA4	gap junction protein, alpha 4, 37kDa	-1.61	6.26E-03	NM_008120
CAPG	capping protein (actin filament), gelsolin-like	-1.81	9.88E-04	NM_007599
CCL6	chemokine (C-C motif) ligand 6	-1.81	6.21E-04	NM_009139
CYBB	cytochrome b-245, beta polypeptide	-1.81	1.02E-02	NM_007807
LRG1	leucine-rich alpha-2-glycoprotein 1	-1.81	2.02E-05	NM_029796
CLEC6A	C-type lectin domain family 6, member A	-1.91	4.42E-03	NM_020001
CLEC7A	C-type lectin domain family 7, member A	-1.91	1.54E-03	NM_020008
ITGB2	integrin, beta 2 (complement component 3 receptor 3 and 4 subunit)	-1.91	2.56E-02	NM_008404
LCN2	lipocalin 2	-2.21	6.12E-05	NM_008491
CCL9	chemokine (C-C motif) ligand 9	-2.81	1.40E-06	NM_011338

[a] Genes with a fold change greater than 1.5 or more ($P<0.05$) of experimental group over controls.

[b] GenBank accession number.
