# Supplementary material for: Bordetella pertussis Infection Exacerbates Influenza Virus Infection through Pertussis Toxin-Mediated Suppression of Innate Immunity
Source: PLoS One. 2011 Apr 20;6(4):e19016. doi: 10.1371/journal.pone.0019016 (PMC3080395; doi:10.1371/journal.pone.0019016)
Supplement: Table S3 — Genes differentially-regulated by PT pretreatment in influenza virus-infected mice at 36 h post-inoculation. (PDF) [file pone.0019016.s006.pdf]

Table S3. Genes differentially-regulated by PT pretreatment in influenza virus-infected mice at 36 h post-inoculation.

Symbol	Gene Annotation [a]	Fold Change	P-value	Accession [b]
ELN	elastin	1.71	1.71E-05	NM_007925
ZBTB16	zinc finger and BTB domain containing 16	1.71	2.84E-09	NM_001033324
NME2	non-metastatic cells 2, protein (NM23B) expressed in	1.61	4.56E-06	NM_001077529
EIF4EBP2	eukaryotic translation initiation factor 4E binding protein 2	1.51	4.04E-02	NM_010124
SOX4	SRY (sex determining region Y)-box 4	1.51	6.26E-03	NM_009238
CCL5	chemokine (C-C motif) ligand 5	-1.51	1.19E-02	NM_013653
CXCR2	chemokine (C-X-C motif) receptor 2	-1.51	1.06E-02	NM_009909
GPR146	G protein-coupled receptor 146	-1.51	2.50E-03	NM_001038703
HCK	hemopoietic cell kinase	-1.51	6.18E-03	NM_010407
IL18BP	interleukin 18 binding protein	-1.51	5.63E-07	NM_010531
IRGM	immunity-related GTPase family, M	-1.51	2.37E-02	NM_008326
NAMPT	nicotinamide phosphoribosyltransferase	-1.51	1.08E-02	NM_021524
PLAUR	plasminogen activator, urokinase receptor	-1.51	2.70E-04	NM_011113
TGFBI	transforming growth factor, beta-induced, 68kDa	-1.51	4.57E-02	NM_009369
TNFAIP3	tumor necrosis factor, alpha-induced protein 3	-1.51	2.05E-03	NM_009397
VCAM1	vascular cell adhesion molecule 1	-1.51	4.16E-02	NM_011693
CCL9	chemokine (C-C motif) ligand 9	-1.61	2.73E-02	NM_011338
CH25H	cholesterol 25-hydroxylase	-1.61	6.82E-03	NM_009890
DUSP1	dual specificity phosphatase 1	-1.61	1.79E-02	NM_013642
F13A1	coagulation factor XIII, A1 polypeptide	-1.61	3.96E-04	NM_028784
HLA-E	major histocompatibility complex, class I, E	-1.61	4.41E-04	NM_010398
LCP2	lymphocyte cytosolic protein 2 (SH2 domain containing leukocyte protein of 76kDa)	-1.61	3.50E-04	NM_010696
MX1	myxovirus (influenza virus) resistance 1, interferon-inducible protein p78 (mouse)	-1.61	2.17E-02	NR_003508
PLAC8	placenta-specific 8	-1.61	6.18E-03	NM_139198
SLFN5	schlafen family member 5	-1.61	9.74E-06	NM_183201
CD300LF	CD300 molecule-like family member f	-1.71	1.04E-02	NM_145634
CLEC7A	C-type lectin domain family 7, member A	-1.71	1.33E-03	NM_020008
CMPK2	cytidine monophosphate (UMP-CMP) kinase 2, mitochondrial	-1.71	1.82E-03	NM_020557
CXCL10	chemokine (C-X-C motif) ligand 10	-1.71	6.00E-03	NM_021274
IGTP	interferon gamma induced GTPase	-1.71	3.20E-03	NM_018738
ITGAM	integrin, alpha M (complement component 3 receptor 3 subunit)	-1.71	4.00E-02	NM_001082960
TAP1	transporter 1, ATP-binding cassette, sub-family B (MDR/TAP)	-1.71	4.29E-03	NM_013683
ANKRD1	ankyrin repeat domain 1 (cardiac muscle)	-1.81	2.01E-04	NM_013468
APOBEC1	apolipoprotein B mRNA editing enzyme, catalytic polypeptide 1	-1.81	1.43E-02	NM_031159
CA13	carbonic anhydrase XIII	-1.81	2.43E-02	NM_024495
GBP2	guanylate binding protein 2, interferon-inducible	-1.81	9.92E-03	NM_010260
HSPA8	heat shock 70kDa protein 8	-1.81	4.48E-08	M13967

IL1RN	interleukin 1 receptor antagonist	-1.81	1.17E-03	NM_031167
IFI44L	interferon-induced protein 44-like	-1.91	3.23E-02	NM_031367
IFIT2	interferon-induced protein with tetratricopeptide repeats 2	-1.91	1.20E-02	NM_008332
IIGP1	interferon inducible GTPase 1	-1.91	4.25E-04	NM_021792
LILRA6	leukocyte immunoglobulin-like receptor, subfamily A (with TM domain), member 6	-1.91	5.83E-04	NM_011088
OAS3	2'-5'-oligoadenylate synthetase 3, 100kDa	-1.91	4.30E-02	NM_145226
PFKFB3	6-phosphofructo-2-kinase/fructose-2,6-biphosphatase 3	-1.91	7.03E-03	NM_133232
CD274	CD274 molecule	-2.01	1.00E-02	NM_021893
THBS1	thrombospondin 1	-2.01	2.65E-03	NM_011580
FCGR1A	Fc fragment of IgG, high affinity Ia, receptor (CD64)	-2.11	7.65E-03	NM_010186
FGL2	fibrinogen-like 2	-2.11	4.31E-03	NM_008013
GBP5	guanylate binding protein 5	-2.11	1.54E-05	NM_153564
SELP	selectin P (granule membrane protein 140kDa, antigen CD62)	-2.11	2.98E-02	NM_011347
RSAD2	radical S-adenosyl methionine domain containing 2	-2.21	5.10E-03	NM_021384

[a] Genes with a fold change greater than 1.5 or more (P<0.05) of experimental group over controls.

[b] GenBank accession number.
